# Correction: Farshadmand et al. Utility of Cardiac Power Hemodynamic Measurements in the Evaluation and Risk Stratification of Cardiovascular Conditions. *Healthcare* 2022, *10*, 2417

**DOI:** 10.3390/healthcare11162323

**Published:** 2023-08-17

**Authors:** Jonathan Farshadmand, Zachary Lowy, Ofek Hai, Roman Zeltser, Amgad N. Makaryus

**Affiliations:** 1 Donald and Barbara Zucker School of Medicine at Hofstra/Northwell, Hofstra University, 500 Hofstra Blvd., Hempstead, NY 11549, USA; 2 Department of Cardiology, Nassau University Medical Center, Hempstead, NY 11554, USA

Zachary Lowy was not included as an author in the original publication [1]. The corrected Author Contributions statement appears here. The authors state that the scientific conclusions are unaffected. This correction was approved by the Academic Editor. The original publication has also been updated.

## References

[1] Farshadmand J., Lowy Z., Hai O., Zeltser R., Makaryus A.N. (2022). Utility of Cardiac Power Hemodynamic Measurements in the Evaluation and Risk Stratification of Cardiovascular Conditions. Healthcare.

